# Instruction effects on randomness in sequence generation

**DOI:** 10.3389/fpsyg.2023.1113654

**Published:** 2023-03-22

**Authors:** Maja Guseva, Carsten Bogler, Carsten Allefeld, John-Dylan Haynes

**Affiliations:** ^1^Bernstein Center for Computational Neuroscience, Charité - Universitätsmedizin Berlin, Berlin, Germany; ^2^Berlin School of Mind and Brain, Humboldt-Universität zu Berlin, Berlin, Germany; ^3^Department of Psychology, Humboldt Universität zu Berlin, Berlin, Germany; ^4^Department of Psychology, City University of London, London, United Kingdom; ^5^Berlin Center for Advanced Neuroimaging, Charité - Universitätsmedizin Berlin, Berlin, Germany

**Keywords:** random number generation, sequence generation, randomness, sequential dependency, free choice, entropy, strategy

## Abstract

Randomness is a fundamental property of human behavior. It occurs both in the form of intrinsic random variability, say when repetitions of a task yield slightly different behavioral outcomes, or in the form of explicit randomness, say when a person tries to avoid being predicted in a game of rock, paper and scissors. Randomness has frequently been studied using random sequence generation tasks (RSG). A key finding has been that humans are poor at deliberately producing random behavior. At the same time, it has been shown that people might be better randomizers if randomness is only an implicit (rather than an explicit) requirement of the task. We therefore hypothesized that randomization performance might vary with the exact instructions with which randomness is elicited. To test this, we acquired data from a large online sample (*n* = 388), where every participant made 1,000 binary choices based on one of the following instructions: choose either randomly, freely, irregularly, according to an imaginary coin toss or perform a perceptual guessing task. Our results show significant differences in randomness between the conditions as quantified by conditional entropy and estimated Markov order. The randomization scores were highest in the conditions where people were asked to be irregular or mentally simulate a random event (coin toss) thus yielding recommendations for future studies on randomization behavior.

## 1. Introduction

Many cognitive tasks could potentially benefit from being able to use randomness, such as exploration (Wilson et al., 2014), predator evasion (Humphries and Driver, 1970), improvisation (de Manzano and Ullén, 2012), creativity (Benedek et al., 2012) or breaking a decision deadlock (see Icard, 2019 for a discussion). The lack of unpredictability in the form of behavioral stereotypy is also a marker of psychopathology (Horne et al., 1982). Due to its relevance for central executive cognitive function, human randomization has received broad attention in the psychology literature (Baddeley, 1966; Miyake et al., 2000; Nickerson, 2002; Oomens et al., 2015).

Traditionally, randomization performance has been assessed by random sequence generation (RSG) tasks where participants are required to make a series of random choices from a predetermined set of options (see Nickerson, 2002). Commonly used choice sets are the digits from 0–9 (Joppich et al., 2004), binary sets, e.g., 0/1 or heads/tails (Nickerson and Butler, 2009), letters A-I (Jahanshahi and Dirnberger, 1999), nouns (Heuer et al., 2010) or symbols on Zener cards (Sisti et al., 2018). However, experimental parameters have varied considerably throughout different RSG versions, including especially the way participants were instructed to be random (Wagenaar, 1972). We hypothesize that this could be a crucial but commonly overlooked moderator of randomization performance.

A common finding of RSG studies is that people are bad randomizers (Nickerson, 2002, see below). Interestingly, if randomness is not overtly requested but rather an implicit requirement, as in competitive games such as matching pennies, randomness seems to be higher (Budescu and Rapoport, 1992). The purpose of this study is to investigate whether randomization performance is affected by how (and especially how overtly) randomness is instructed for, by directly comparing different instructions in an otherwise identical experimental setting.

## 2. What is behavioral randomness?

Human randomization performance is typically assessed with some form of RSG task, where a participant is asked to produce a series of random choices from a prespecified choice set in an unpredictable way (Nickerson, 2002). In the field of psychology there are two related aspects of behavioral randomness, randomness production and randomness perception. In this article we focus on the first, the way people produce a random sequence of choices. Here we encounter multiple methodological challenges.

First, randomization can be described in two ways, by focusing on the random process or the random product, i.e., an individual finite sequence (Nickerson, 2002). A specific finite sequence could have been generated by a random process, but might not appear random, e.g., a sequence of 10 heads in a row can be created by a fair coin toss. Alternatively, a finite sequence can appear random but it could have been produced in a deterministic, predictable way, such as the digits of number pi or pseudo-generated sequences used in computers (Nickerson, 2002). In our study we can only observe the behavioral outcome directly, which is the sequence of choices produced by humans. Statements on the process that brought about the outcome will ultimately be of speculative nature (see also next section).

Even after narrowing the scope to the observable product of randomization, it remains difficult to conceptualize what it actually means to say that this product, e.g., a choice sequence, is random. Generally, there are different angles from which a random sequence can be characterized, e.g., by referring to aspects of incompressibility, irregularity, or equiprobability (see Nickerson (2002) for a review). Here we focus on sequential independence, which states that a choice at any given time point is independent of preceding time points and likewise has no bearing on the subsequent choices. In section 6.4.3. on randomness measures we present a more detailed reflection and description on how we capture aspects of randomness by identifying sequential independencies borrowing from Markov Chain Theory. In the following, whenever we talk about people “randomizing,” we mean attempting to randomize according to their own definition of randomness.

## 3. Randomization and executive control

The ability to generate random behavior is cognitively demanding (Jahanshahi et al., 2006), but it can be highly useful and adaptive. Examples range from protean strategies, i.e., to conceal intentions to evade predators (Humphries and Driver, 1970; Tervo et al., 2014; Icard, 2019; Belkaid et al., 2020), to mixed strategies, where randomizing over choice options in a game setting is a Nash equilibrium (Platt, 2004; Lee and Seo, 2011). Behavioral randomness also plays a role in random exploration to discover new foraging opportunities in dynamic environments (Jensen, 2018; Belkaid et al., 2020), the emergence of creative thought (Campbell, 1960), and the mating behavior of songbirds (Jensen, 2018). Not only is the capacity for random behavior itself functional, but also the overarching ability to successfully identify and calibrate when to use randomization based on the given environmental demands: “People and animals choose whether, when, and how much to vary […].” (Neuringer, 2002, p. 672; see also Neuringer and Jensen, 2010).

Accomplishing this complex task requires the coordination of different cognitive processes, which fall under the umbrella of executive functions. Executive functions are a set of higher order cognitive control processes that are active in planning or problem-solving where attention is required because they cannot be performed automatically (Cristofori et al., 2019). Studies using the RSG task have shown that randomization draws on several executive subfunctions such as the inhibition of prepotent responses, maintenance of choice history in working memory and updating of response strategies (Baddeley et al., 1998; Jahanshahi and Dirnberger, 1999; Miyake et al., 2000).

A few mechanistic models of RSG have been proposed. In Baddeley et al.’s (1998) verbal model a response (“schema”) is selected from a set of possible responses, e.g., “decrease previous response by 1″ and evaluated relative to the running record of previous responses in working memory. If the schema fits one’s notion of randomness, the selected response is produced, else it is inhibited and a new response is selected. The inhibitory aspect of RSG was further developed by Jahanshahi et al. (1998) in their “network modulation model.” Here the elements in a given choice set (e.g., numbers 0–9) are linked together in a semantic network where link weights are determined by learned associations (e.g., adjacent numbers), representation strength (e.g., 1 tends to appear more often as a first digit, see the law of Benford’s, 1938). As these weights bias the response selection (randomness would arise from equal weights), a limited-capacity executive controller needs down-regulating stronger weights, thereby inhibiting the selection of, e.g., adjacent numbers. Additionally, the controller is blocking immediately preceding numbers (“refractory mechanism”) to avoid repetition (Jahanshahi et al., 1998).

Shortcomings in any of the executive subfunctions result in biased, nonrandom sequences. For example, weak inhibitory performance presents itself in the form of a seriation bias, which is the propensity to produce stereotypical overlearned responses, such as counting upwards. In addition, a deficient ability in updating typically leads to the cycling bias, where the participant cycles through every possible option in the choice set before repeating (Rabinowitz, 1970; Ginsburg and Karpiuk, 1994; Peters et al., 2007). Another pervasive bias is the avoidance of repetitions, also called *negative recency effect* or *alternation bias*, where an excess of alternations and suppression of repeating choices can be observed (Falk and Konold, 1997).

Given the strong role of executive functions in randomization, there have been a number of studies showing that randomization performance is compromised in various psychopathologies that have been discussed to also affect executive processes (Horne et al., 1982). For example, there is a tendency for responses to be stereotypical in disorders such as schizophrenia (Mittenecker, 1960; Chan et al., 2011), Parkinson’s disease (Spatt and Goldenberg, 1993), dementia of the Alzheimer type (Brugger et al., 1996), aphasia (Proios et al., 2008) or multiple or sclerosis (Geisseler et al., 2016). But also transient variations in executive functions in healthy subjects such as those induced by sleep deprivation (Heuer et al., 2005) or working memory load manipulations in dual task settings (Naefgen and Janczyk, 2018) affect randomization performance.

## 4. The role of instructions in randomization tasks

A comprehensive review by Wagenaar (1972) highlighted a key problem in the RSG literature, which is the high variability of task parameters across studies. For example, the number of options in the choice set, the nature of the choice set (numbers, letters or buttons), sequence length, whether responses were paced (e.g., 1 choice per second) or not, and visibility of previous responses, to name a few.

There is also considerable variability across studies in the way random behavior is instructed. In some experimental versions participants were simply asked to make random choices (Oomens et al., 2015; Sisti et al., 2018), others used analogies and mental models to illustrate the concept of randomness, such as the hat analogy, where one imagines to blindly draw pieces of paper with numbers written on them out of a hat, reads them out loud and puts them back in the hat (Joppich et al., 2004). Also often used were die or coin analogies, where the instructions were to report the results of an imagined die (Knoch et al., 2004) or coin toss (Nickerson and Butler, 2009). A different way of instructing has been to encourage unpredictability by avoiding “schemes” or patterns (Daniels et al., 2003). For example, Azouvi et al. (1996) encouraged a “completely jumbled” sequence, without consecutive digits like “1-2-3-4-5,” or as instructed in Finke (1984): “in such a manner that if another person were trying to predict which number would be selected next, he or she would not be able to do so” (p. 40).

The above-mentioned instruction types evoke some notions of randomness by either mentioning the word random or by means of an analogy. In this way behavioral randomness becomes an active goal in the task. Alternatively, there are task types where randomization can emerge without any reference to randomness or a randomization process. One example is to appeal to the concept of spontaneity in free choice tasks (Naefgen and Janczyk, 2018). The idea is that people do something similar to an RSG task when prompted to decide freely between options, which is why tasks related to RSG are frequently used in the volition literature for Libet-style experiments (Soon et al., 2008; Bode et al., 2011; Lages and Jaworska, 2012; Lages et al., 2013). Further, several studies suggest that there is an overlap between brain activity in free choice tasks and RSG tasks (Frith et al., 1991; de Manzano and Ullén, 2012). However, free choice tasks typically still constrain the participants to answer in a specific way, such as avoiding patterns and balancing the amount of responses (Goldberg et al., 2008; Naefgen and Janczyk, 2018), thus calling into question whether the responses are actually subjectively free.

An alternative paradigm to covertly elicit random behavior is by means of a zero-sum game, e.g., matching pennies game. In this game the best response of each player to each other (Nash equilibrium is attained when both play a mixed strategy which means choosing each option with a probability of 0.5 (Nash, 1950; Lee and Seo, 2011)). Comparing the choice sequences obtained from a matching pennies game to those from a standard RSG task on a variety of different randomness measures (see below for a discussion), Budescu and Rapoport (1992) found a better randomization performance in the first than in the latter.

Another case where choice sequences exhibit similar structural characteristics as those reported in RSG, is in perceptual judgment tasks (Bode et al., 2013; Urai et al., 2019). For instance, although any low-level perceptual decision should be based solely on the given stimulus content, i.e., history-independent, there is a wealth of studies showing the presence of sequential dependencies in choice sequences, where an individual’s history of perceptual decisions was shown to bias the subsequent decision (Cicchini et al., 2018; Gallagher and Benton, 2022).

A similar guessing paradigm is the Zener card test where participants have to purely guess one of five symbols on a card that the researcher is covertly holding in their hand. Since the symbols are neutral, occur with the same probability and participants receive no feedback on the correctness of their choices, there should be no preference for choosing any of the symbols which would essentially liken the Zener card test to a perceptual judgment task without perceptual information. As such, the choice sequences produced in this test exhibit similar biases as perceptual judgment and RSG tasks, such as repetition avoidance (Sisti et al., 2018).

Apart from RSG it is well known that task instructions are a powerful way of modifying behavior (Dreisbach and Haider, 2008; Gaschler et al., 2012; Schneider and Logan, 2014). However even in the absence of explicit task instructions seemingly moderate changes in instruction wording can cause large shifts in behavior (Burnham et al., 2000; Cooper et al., 2001; Rahhal et al., 2001; Liberman et al., 2004; Smilek et al., 2006; Gilbert et al., 2009; McCabe and Geraci, 2009, see also table 1 in Oppenheimer et al. (2009) for an overview). In these experiments people responded very differently to the same materials and tasks based only on small differences in instruction wording. This phenomenon is also known as the “framing effect” and well-documented in behavioral and experimental economics (see Levin et al. (1998) for a review). A prominent example is that by solely naming the prisoners’ dilemma game either “WallStreet Game” or “Community Game” has an impact on cooperation/defection outcomes in players (Liberman et al., 2004). While the behavioral and experimental economics literature has since established a need for clear, decontextualized and standardized rules in which tasks such as games are explained to participants, this has not been the case in the RSG literature as evidenced by the task instruction variations described above.

**Table 1:** Wording of instructions.

Condition	Instruction
Explicit Randomness (*ER*)	“You have to choose the sides of the coin randomly.”
Free Choice (*FC*)	“There is no right or wrong answer, we want you to decide spontaneously. Which side you choose in every trial is your own free choice.”
Irregularity (*IR*)	“You have to select the sides in a maximally irregular and chaotic way. In other words, someone who would look at your sequence of choices, should not be able to see any pattern or regularity.”
Mental Coin Toss (*MC*)	“You have to simulate a coin toss in your head and choose the side that came up. The goal is to produce a sequence of choices that is not different from the results of a real fair coin toss.”
Perceptual Guessing (*PG*)	“You will have to indicate which of the coins that you saw was darker.”

## 5. Comparison of five different RSG task instructions

All these findings together suggest that randomization performance is influenced by the details of the instructions. Thus, in the present study we constructed five different conditions where participants were asked to choose either (1) randomly, (2) freely, (3) irregularly, (4) according to an imagined coin toss, or (5) according to a perceptual guessing task (for full details see below). Our goal was to examine how the different ways of instructing participants affect the randomness of the generated sequences. Following the terminology of Brass and Haggard (2008), the tasks would constitute a “What” decision.

The conditions vary in the degree to which the goal of producing a random sequence is made explicit. At one end, there is only one condition where we explicitly ask to make a random choice while at the other end, there is the perceptual guessing task, where randomness is not intended but may occur incidentally. The other instruction conditions fall somewhere in between these two extremes. For example, in the mental coin toss condition some level of randomness is presumably considered essential by the participant to emulate the physical process. In none of our conditions was the randomness explicitly required to “win” a game as in competitive interaction games.

Importantly, we kept every other fundamental feature in the experiment identical, such as the choice set size, length of time window for making the decisions, sequence length, etc., thereby addressing the heterogeneity of experimental versions of the RSG task in the literature (Wagenaar, 1972) and allowing proper comparability across conditions.

Our primary statistical hypothesis was that there is a difference in the degree of randomness between the sequences generated in the five conditions. Besides this primary hypothesis there are many reasons that a specific instruction could either increase or decrease the randomization performance compared to the others. These are discussed in the following.

First, if it matters how overtly the concept of randomness is expressed, we expect a worse performance in the condition where randomness is explicitly instructed for and a better performance in a condition where randomness is not mentioned at all, such as in the guessing task where random behavior might be elicited as an incidental byproduct.

Second, our conditions also differ by the propensity to hold the choice history in mind. We expect that participants who were asked to choose freely or who completed the guessing task should feel no need in keeping a tally of previous responses on each trial. But somebody who is, e.g., instructed to create an irregular sequence might be monitoring the previous responses in order to assess whether they follow the instructions. Consequently, this monitoring process would cause additional working memory load. The direction in which the additional working memory load will affect however is not so clear. Empirically, we know from previous dual task paradigms studies that higher working memory load decreases RSG performance (Cooper et al., 2012; Naefgen and Janczyk, 2018). Based on this consideration we expect randomization performance to be worse in those conditions where monitoring processes consume working memory capacity such as in the irregularity condition as well as mental coin toss and explicit randomness tasks. At the same time, randomness as it is assessed here is per definition a memoryless process because it involves sequential independence (see section 2. What is behavioral randomness?). This implies that a completely depleted working memory should benefit randomization producing the opposite expected outcome.

At the same time, these conditions possibly force the participants to actively suppress the urge to answer in a patterned, predictable way. According to Wegner’s (1994) ironic process theory whenever people actively try to avoid thinking a specific thought, it ironically occurs more often than it would without the conscious effort to suppress it (also known as immediate enhancement effect (Wang et al., 2020)). If the effort to suppress creates the opposite effect, we might even see a better randomization performance in the free choice and guessing task where there is no reason to engage in suppression. Finally, the explicit instruction to be random depends on the subjects’ individual concepts of randomness. However, instructing somebody to be irregular or throw a coin in the head is arguably a more direct and actionable instruction that might allow for less space for misunderstanding.

## 6. Methods

### 6.1. Procedure

The participants accessed the link to the study *via* the Prolific platform where they were first informed about the experimental procedure and required to declare their consent. Then, after being randomly assigned to one of the five experimental conditions, the respective instructions were presented. After reporting the stimulus screens, we proceed with outlining the different instruction types. In the beginning participants had the opportunity to practice the task. To make sure that the instructions were read and understood, we implemented a brief quiz with 5 questions pertaining to the different stages of a trial as well as the overall nature of the task.

**Figure 1:**
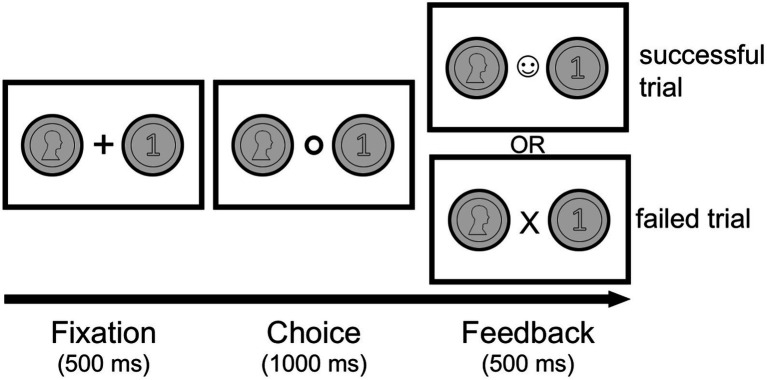
Example trial in the experiment. After a brief fixation phase (500 ms) follows the decision phase (1,000 ms), where the participant has to make a choice using the left or right keyboard arrow. The subsequent feedback phase (500 ms) indicates the timing of the button press. If the key is pressed on time, the circle turns into a smiley (successful trial). If the key is pressed too late, the circle turns into an X (failed trial).

Figure 1 shows the experimental procedure. Each trial began with a presentation of the front (heads) and backside (tails) of a coin (500 ms) to both sides of a fixation cross in the center. The positioning of the coin on the left or right side was pseudo-randomized between participants and kept fixed throughout the whole experiment within each participant. As soon as the fixation cross turned into a circle, subjects had 1,000 ms to indicate their choice by pressing the left or right keyboard arrow with their right hand. If they succeeded to press the arrow in the allotted time, the circle changed to a smiling face. If they failed, they saw an “X” instead (please note: the feedback is not linked to the specific choice). After this feedback (500 ms) the fixation cross appeared again and a new trial began. This procedure was identical across all conditions except for a small difference in the *Perceptual Guessing* condition (see below), where the coin images were only shown in the fixation phase and disappeared in both the choice and feedback stages.

In order to avoid carry-over effects between conditions, the experiment was conducted as a between-subject study with five different instructions (one per group): *Explicit Randomness* (ER), *Free Choice* (FC), *Irregularity* (IR), *Mental Coin Toss* (MC), *Perceptual Guessing* (PG). These conditions differed in the way participants were instructed to generate the sequence.

In the *Explicit Randomness* condition, we explicitly instructed subjects to choose randomly. The *Free Choice* instruction required to spontaneously choose any side of the coin in an unconstrained manner. In the *Irregularity* condition we instructed participants to create a sequence that was maximally irregular. The instructions in the *Mental Coin Toss* condition were to mentally simulate a sequence that was indistinguishable from a truly random process, i.e., a coin toss. The *Perceptual Guessing* condition involved a perceptual decision-making task, where the two conditions that had to be identified were physically identical. Specifically, the participant was shown the same two coins as in the other conditions and was asked to indicate which of the coins was darker in tonal value. Unbeknownst to the participant there was no actual difference in tonal values between the two coin images, which meant that the participant had to make a random guess. See Table 1 for the exact wording used in the experiment.

To ensure a balanced sequence length across all subjects, failed trials were not counted by adding another trial to the end of the block. Thus, each participant completed 1,000 trials in total, with 200 trials per each of the five blocks. There was a two-minute break between each block when we presented two questions “How focused was your attention during this block?” and “How closely did you follow the instructions?” on a five-point Likert scale.

After the main task we repeated the quiz by once again asking what the task was. In the final part they had to describe their choice strategy in an open-ended form as well as indicate whether and when their strategy changed over the course of the study. Additionally, we debriefed those who completed the *Perceptual Guessing* condition about the actual task and asked whether they had realized that the coins were identical in tonal value. Participation in the experiment was compensated with 7€ per hour. The experimental procedure was approved by the Ethics Committee of the Department of Psychology (Humboldt University of Berlin) in accordance with the Declaration of Helsinki.

### 6.2. Participants

Invitations were sent out *via* the online recruitment service Prolific^1^ to the participant pool, which was restricted to those who self-reported to not have any diagnosed, on-going mental health conditions. Within this subject pool, participants would be admitted on a first-come-first-served basis.

An initial sample size estimation was performed with G*Power 3.1 (Faul et al., 2007). The calculations were based on a medium effect size (Cohen’s *f* = 0.25 (Cohen, 1988)), type I error rate alpha = 0.05, statistical power (1 - beta) = 0.8 and 5 groups which resulted in a total sample size of 200 participants. Given the pervasive problem of underpowered studies in the psychology literature (Szucs and Ioannidis, 2017) and the ease of online data collection we decided to double the sample size.

A total of 543 participants completed the online experiment. Of these participants, 72 failed at least one of our five *a-priori* established exclusion criteria (see “Data analysis plan” section below). Of the remaining participants, 62 people in the *Perceptual Guessing* condition failed the manipulation check, meaning that they realized that the coins were the same tonal value. 5 datasets had to be discarded due to corrupted files. 16 datasets were removed because these participants always pressed the same button in at least one block. These excluded participants had been allocated to the following conditions: 3 in *Explicit Randomness*, 11 in *Free Choice*, 2 in *Mental Coin Toss*. Finally, one person was removed because they reported typing a text in morse code during the task (*Free Choice* condition).

Excluding all these people, we were left with a final sample of 388 participants aged between 18 and 69 with a mean age of 27 (*SD* = 8.28). In this sample, 277 participants self-reported as male, 111 self-reported as female as their assigned sex at birth (binary closed-format question). None of the subjects have been diagnosed with a psychiatric condition. 203 of the 388 participants indicated a student status. Participants on the online platform receive a reputation score that summarizes how often a person was rejected in past experiments by the researchers. The score ranges from 0–100, where the lower the number the higher the rejection rate in the past. The participants in our sample have a mean score of 99.4 (*SD* = 1.6), which is reassuring in terms of submission quality. Table 2 shows a breakdown of the sample characteristics across each condition.

**Table 2:** Sample characteristics.

Condition	*n*	Age M SD	% Female	% Student status	Prolific reputation score M SD
Explicit Randomness (*ER*)	80	26.7 (9.4)	26.3	50.0	99.4 (1.5)
Free Choice (*FC*)	73	27.7 (8.2)	28.8	52.1	99.6 (1.2)
Irregularity (*IR*)	80	25.9 (7.3)	27.5	58.8	99.0 (2.4)
Mental Coin Toss (*MC*)	85	27.3 (8.1)	30.6	51.8	99.5 (1.2)
Perceptual Guessing (*PG*)	70	27.1 (8.3)	30.0	48.6	99.7 (0.7)

### 6.3. Materials

The study was written with jsPsych (de Leeuw and Motz, 2016), a JavaScript library which was created for web browser-based behavioral testing. It was then deployed and hosted on our own university servers using JATOS (Lange et al., 2015). Participants performed the experiment on their own devices. Participation was possible only on a desktop computer or laptop, no mobile or tablet devices were allowed. Further, participants were instructed to use Firefox, Chrome or Opera browsers with at least 1,000 × 700 px window size. This was a binding requirement without which the experiment would not start. We asked the participants to perform the experiment in a calm and distraction-free environment. The experiment could be aborted at any time by clicking on a button in the bottom of the screen which immediately deleted the participants’ data from our servers. Importantly, this would not affect the participant’s reputation score on Prolific.

### 6.4. Data analysis

#### 6.4.1. Exclusion

In an online study it is especially difficult to ensure that participants fully understand and comply with the task and that they concentrate on its completion. To identify low quality data sets, we implemented a series of attention checks that were defined *a-priori* in the preregistration. The number of data sets rejected in each exclusion category is indicated in parentheses below.

First, we examined how much time participants dedicated to reading the instructions and excluded any submission with less than 60 s reading time (10 submissions). Second, we took into account the answers that participants gave in the brief quiz in the beginning of the study. To qualify all but two questions had to be answered correctly in a first try (6 submissions) and all questions had to be answered correctly in a second try (22 submissions). The quiz was not difficult and we expected that a reasonably diligent participant would be capable of answering all of them correctly after having read the instructions and performed the practice trials. Third, after the main experiment we probed participants again about the nature of the task, which had to be answered correctly (7 submissions). Fourth, we excluded submissions with more than 15 successively failed trials in any of the five blocks (39 submissions) or more than 50 failed trials over all blocks (34 submissions). And finally, we recorded browser interactions, i.e., information on whether and how long a person changed to a tab or a window that was not related to the experiment or exited the full screen. We excluded any submission with more than three instances of browser interactions during either of the five blocks (18 submissions).

#### 6.4.2. General statistical properties

Before conducting the main analysis, we computed the statistical properties of the sequences. These measures were also calculated from a simulated set of 78 binary sequences of length 1,000 digits each, created with Matlab’s pseudorandom generator using the default “Mersenne twister” algorithm, in order to allow for comparisons. We chose to simulate 78 sequences to match the average sample sizes across all five conditions.

First, we assessed how *balanced* the sequences were by calculating the proportion value, i.e., the relative frequency of heads to tails decisions per block. Since the position of the heads and tails coins was randomized across the participants, we entered the higher number of the respective options in the numerator, i.e., we calculated $\frac{\max\left(\left[numberofheads\right],\left[numberoftails\right]\right)}{n\;trials}$. A proportion value of 0.5 means that both left and right button presses occurred with equal frequency, i.e., 100 times each within a block. A proportion value of 1 means that only one of the two options was chosen throughout the block.

Second, we determined the average *run length* per person. A run is an uninterrupted sequence of identical choices. For example, in the sequence *H-T-T-H-H-H-T* there are two runs of length 1 (*H* and *T*), one run of length 2 (*T-T*) and one run of length 3 (*H-H-H*). Note that there is a degree of uncertainty regarding the length of the last run because this is a truncated observation; we do not know whether this run would have continued if we would not have stopped the block. This is why we omitted the last run in each of the 5 blocks.

#### 6.4.3. Randomness measures

Randomness is difficult to define and to measure in general (Volchan, 2002). One of the issues is that it is not possible to create a test that conclusively proves randomness, as this requires a demonstration that no regularity exists in the sequence, which is essentially a proof of a null hypothesis (Ayton et al., 1989). Moreover, violations of randomness could in principle occur in an infinite number of different ways, including strategies that are very hard to detect; if say a participant knew the digits of pi by heart and would use them to determine the choice on each trial. Consequently, we can only use tests that detect specific deviations from randomness, or specific forms of regularity. These deviations have been captured throughout the RSG literature with different sets of tests and measures, such as the runs test, digram frequency, count score or turning point index to name a few (see Ginsburg and Karpiuk (1994) and Towse and Neil (1998) for details).

**Figure 2:**
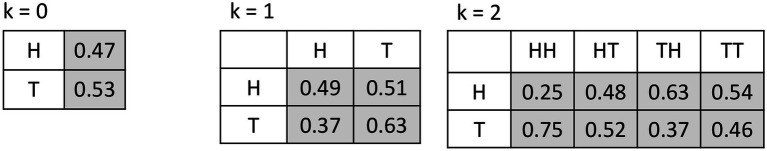
Example transition probability matrices for orders *k* = 0, 1, and 2. For the 0th order, there is no dependency on previous choices, and the two values represent the probabilities for the subsequent choice to be “H” or “T.” For higher order, each column corresponds to a possible sequence of previous choices and gives the probabilities for the subsequent choice under the condition that that sequence occurred. For example, in *k* = 2, after the choice pair “H-H,” the probability to choose a “T” is three times as large as the probability to choose another “H.”

A problem with these diverse measures is that it is usually unclear why a specific randomness measure was chosen in a study. While these measures certainly tap into various separate aspects of randomness vs. regularity, they lack a theoretical motivation. In this study we decided to adopt the perspective of stochastic processes, from which the natural way to describe a sequence of discrete choices is a Markov chain (Cover and Thomas, 2005; Allefeld et al., 2013). A Markov chain is defined by an order *k*, the number of previous choices which affect the subsequent choice, as well as transition probabilities $\mathrm{Pr}\left(X_{t}|X_{t-1},X_{t-2},\ldots ,X_{t-k}\right)$, the probability for each possible subsequent choice given the previousk choices (Figure 2). The assumption underlying a Markov chain model is that sequential dependencies are temporally limited, and the Markov order reflects this temporal extent of memory. The transition probabilities are summarized by the conditional entropy rate at a given order, the natural measure of randomness of a stochastic process. Our approach still picks up many if not all of the regularities targeted by the measures mentioned above, but does so in a parsimonious and theoretically motivated way. Nevertheless, we would like to emphasize that it is still possible that a sequence has an underlying structure which cannot be captured by these metrics. Further, we would like to point out that our approach involves a statistical model, the Markov stochastic process, and does not directly relate to a cognitive mechanism that brings about the behavior.

In the present study, each participant performed a sequence of 1,000 binary choices between heads (“H”) and tails (“T”), broken into five blocks with a brief break in between. We therefore decided to apply the calculations described in the following separately to each contiguous sequence of 200 choices, and use the means across blocks as the per-participant outcomes entering statistical analysis (see next section).

For a given order *k*, we estimated the transition probabilities by maximum likelihood, which results in the following procedure:

- From the observed choice sequence, collect all subsequences of length *k + 1*, $\left(x_{1},x_{2},\ldots ,x_{k},x_{k+1}\right)$, and count them grouped by (a) the last choice $x_{k+1}$ and (b) the initial choice sequence $\left(x_{1},x_{2},\ldots ,x_{k}\right)$, resulting in counts $c\left(x_{k+1},\left(x_{1},x_{2},\ldots ,x_{k}\right)\right)$.
- For each initial choice sequence separately, divide the counts for the two possible last choices by their sum:

$$\frac{c\left(x_{k+1},\left(x_{1},x_{2},\ldots ,x_{k}\right)\right)}{\sum_{x_{k+1}\in \left\{\mathrm{H,T}\right\}}c\left(x_{k+1},\left(x_{1},x_{2},\ldots ,x_{k}\right)\right)}.$$

This provides the estimates of


$$\mathrm{Pr}\left(X_{t}=x_{k+1}|X_{t-1}=x_{k},X_{t-2}=x_{k-1},\ldots X_{t-k}=x_{1}\right).$$

For a sequence of 200 choices, estimation of a kth order Markov chain is based on *200 - k* subsequences of length *k + 1*. The largest order for which the model can be estimated is *k = 199*, though for higher orders the transition probability estimates become more and more imprecise.

The maximum likelihood estimation also results in a maximized likelihood, which quantifies the precision with which the estimated model describes the observed data. However, this quantity is not useful to compare the quality of models of different order, because with increasing order the number of model parameters increases exponentially, so that higher order models always appear to be a better fit for the data than lower order models. To estimate the optimal Markov chain order, we therefore followed the approach of Csiszár and Shields (2000) to use the Bayesian Information Criterion (BIC; Schwarz, 1978), which chooses the best model by the maximized likelihood minus a penalty which depends on the number of parameters. Csiszár and Shields (2000) proved that (in contrast to other criteria) the BIC Markov order estimator is consistent, i.e., with increasing sample size the probability to estimate the true order approaches 1. Our first outcome measure is the BIC-estimated **optimal Markov order**.

The Markov order quantifies the temporal extent of sequential dependencies, i.e., how many previous choices influence the subsequent choice. As the Markov order of a model increases from 0, the subsequent choice becomes more and more precisely determined. In the extreme case of an underlying deterministic process all transition probabilities are either 0 or 1. However, if at the true order (some) transition probabilities remain between 0 and 1, there is an irreducible remaining randomness in the process. Information entropy (Shannon, 1948; Cover and Thomas, 2005; Jensen et al., 2013) quantifies the uncertainty of a signal, for a binary choice between 0 bit (perfectly determined) and 1 bit (perfectly random). For a Markov chain, one considers the entropy of the probability distribution for the next choice conditional on the k previous choices (i.e., the Markov transition probabilities), taking a weighted average across previous choices according to their probability; this is called conditional entropy rate. The result reflects how much uncertainty remains over the subsequent choice, on average. It is called „rate“because it refers to a single step in an ongoing process, reflecting that a Markov process can be seen as continually producing uncertainty (entropy).

The estimation properties of the conditional entropy rate depend critically on the precision with which transition probabilities have been estimated, which in turn depends on the assumed Markov order (see above). In particular, the larger *k*, the larger the number of previous states over which data are split, and therefore the smaller the number of data points available for each conditional probability estimate. This tends to make estimated distributions look less flat and therefore less entropic. If we were to use the BIC-optimal Markov order in each case, because of this estimation bias results would not be comparable across participants and conditions, and an observed difference in the conditional entropy rate could actually just reflect a difference in the optimal order.

To make the measure comparable between participants and conditions, we therefore decided to estimate it at a fixed Markov order of *k = 3*, which is as large as or larger than the estimated optimal order across participants and conditions. It is not a problem to use a k which is larger than the optimal order, because this just means that additionally previous choices are used even though they do not characterize the process more precisely. Consequently, conditional distributions do not lose entropy, and therefore the underlying conditional entropy rate, which is being estimated, is unchanged. Our second outcome measure is thus the **conditional entropy rate** estimated at *k = 3*.

#### 6.4.4. Description of statistical tests

Data processing and statistical analysis were done using Matlab, R2020b (R Core Team, 2020) and RStudio (v.1.3.1093). The materials for this study are available by emailing the corresponding author. Analysis code and data are available under https://osf.io/z8rjx/files. As stated above, we calculated the two outcome variables optimal Markov order and conditional entropy separately over the choice sequences (200 choices) for each of the five blocks and computed the mean over the blocks for each person. We have omitted the trials from the analysis where participants failed to make a choice in time and the sequence analysis was performed on the remaining trials. Please note that this happened only very rarely: On average participants failed to make a choice 7 times (*SD* = 9) across the experiment, which amounts to an average of 0.7% (*SD* = 0.91) of the whole sequence.

Due to the non-normal nature of the summary variables defined above, we performed two separate non-parametric Kruskal-Wallis analyses at a significance level of 0.05 in order to determine whether (a) average optimal Markov orders and (b) conditional entropy values differed between the five conditions (independent variable). In case the null hypothesis of equal means was rejected, *post hoc* tests were performed at a significance level of 0.05 using a Tukey–Kramer adjustment to identify the specific pairs that differed significantly from each other. Effect sizes and their CIs were calculated with R *rstatix* package using *kruskal_effsize* (Kassambara, 2021).

In line with our preregistration report, we also conducted a more detailed multilevel analysis of our data (Hox and Maas, 2005). The goal of this approach was to account for potential fatigue effects that may set in after doing blocks of the rather monotonous task. On the first level a regression model explained the outcome variable by block number (1–5) plus intercept as random effects. This estimated intercept served then as the dependent variable on the second level, where a one-way ANOVA was used to identify differences between the five instruction conditions. This was done separately for optimal Markov order and conditional entropy.

## 7. Results

### 7.1. Attention and instruction adherence

**Figure 3:**
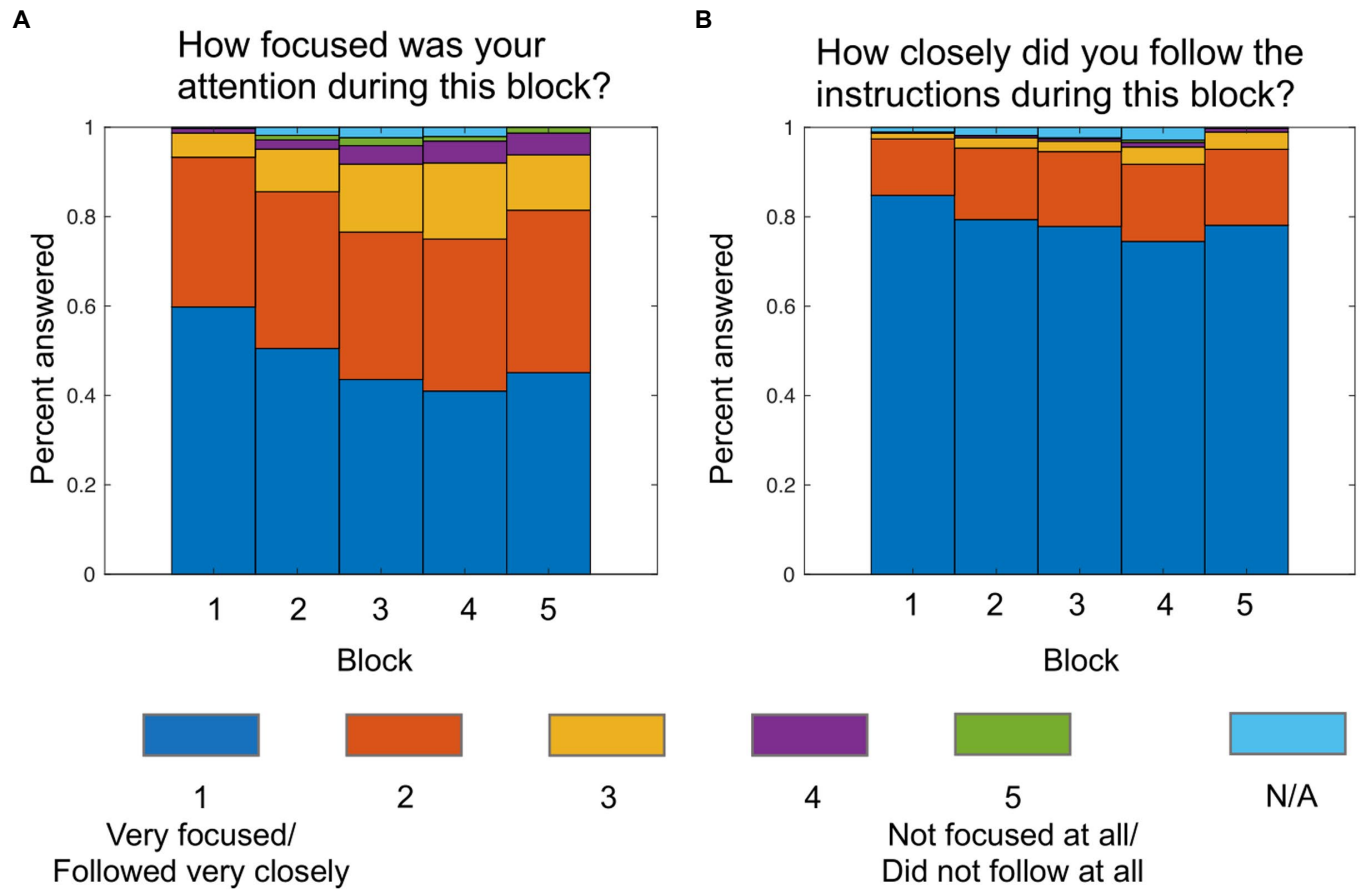
Answers to attention **(A)** and instruction adherence **(B)** questions after each block. N/A values indicate that the question was not answered. This was possible because the experiment automatically continued after the 2-min break was over.

Figure 3 shows the distribution of the answers to the two questions on attention and instruction adherence that were asked at the end of each block. Based on the mode for each participant calculated over the five blocks, 84.8% were “very focused” to “focused” and 96.9% followed the instructions “very closely” to “closely,” indicating a good self-reported level of attention and instruction adherence. 64.4% of participants indicated that they used the same approach throughout the whole experiment.

### 7.2. General statistical properties of sequences

**Figure 4:**
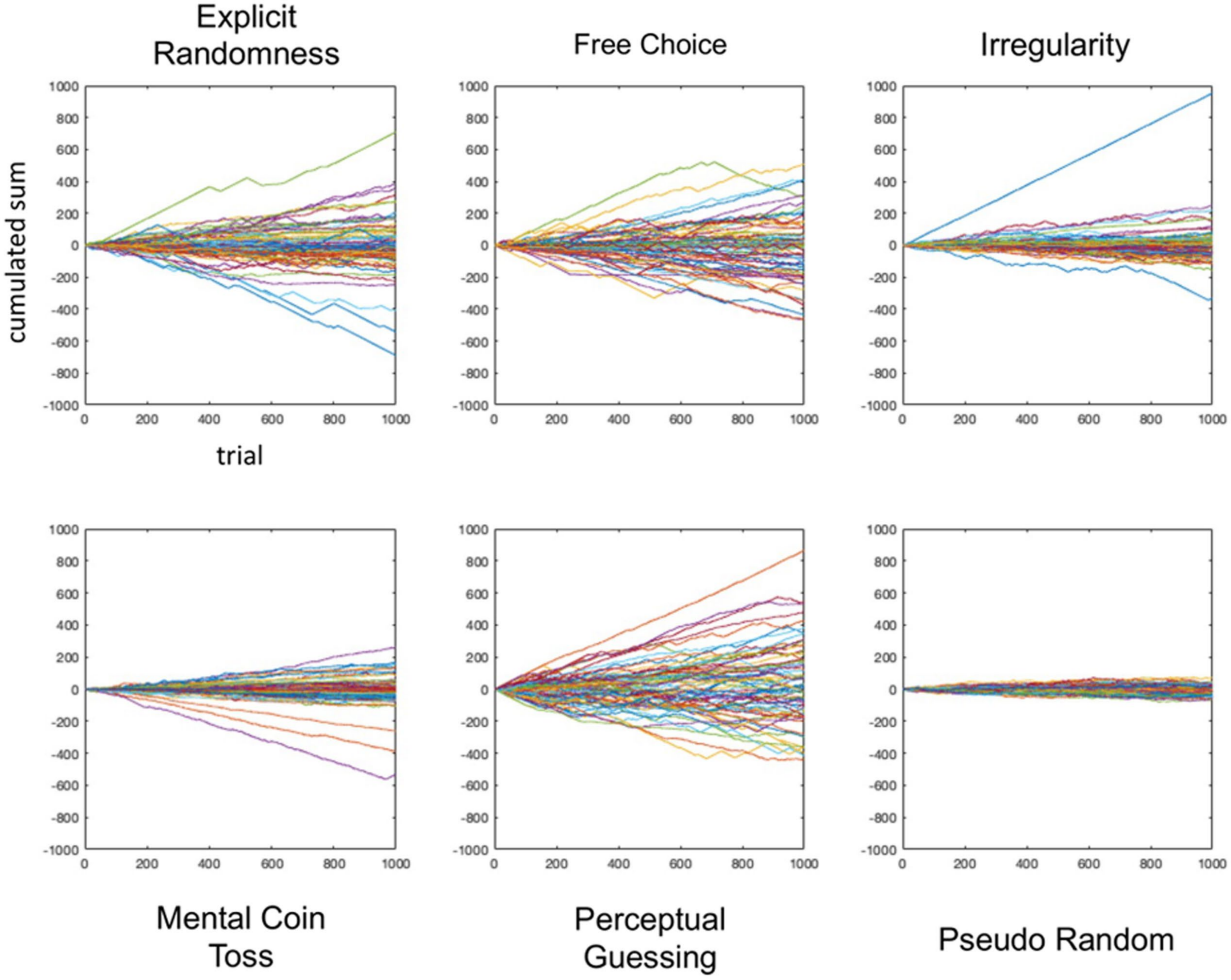
Plot of cumulative sum sequences for each condition. Every line is one participant.

For a first assessment of the time series, we plotted the cumulative sum of the participants’ time series in which we coded “1″ for *tails* and “-1″ for *heads*. Figure 4 shows the series for each of the five conditions as well as the simulated pseudorandom series.

**Figure 5:**
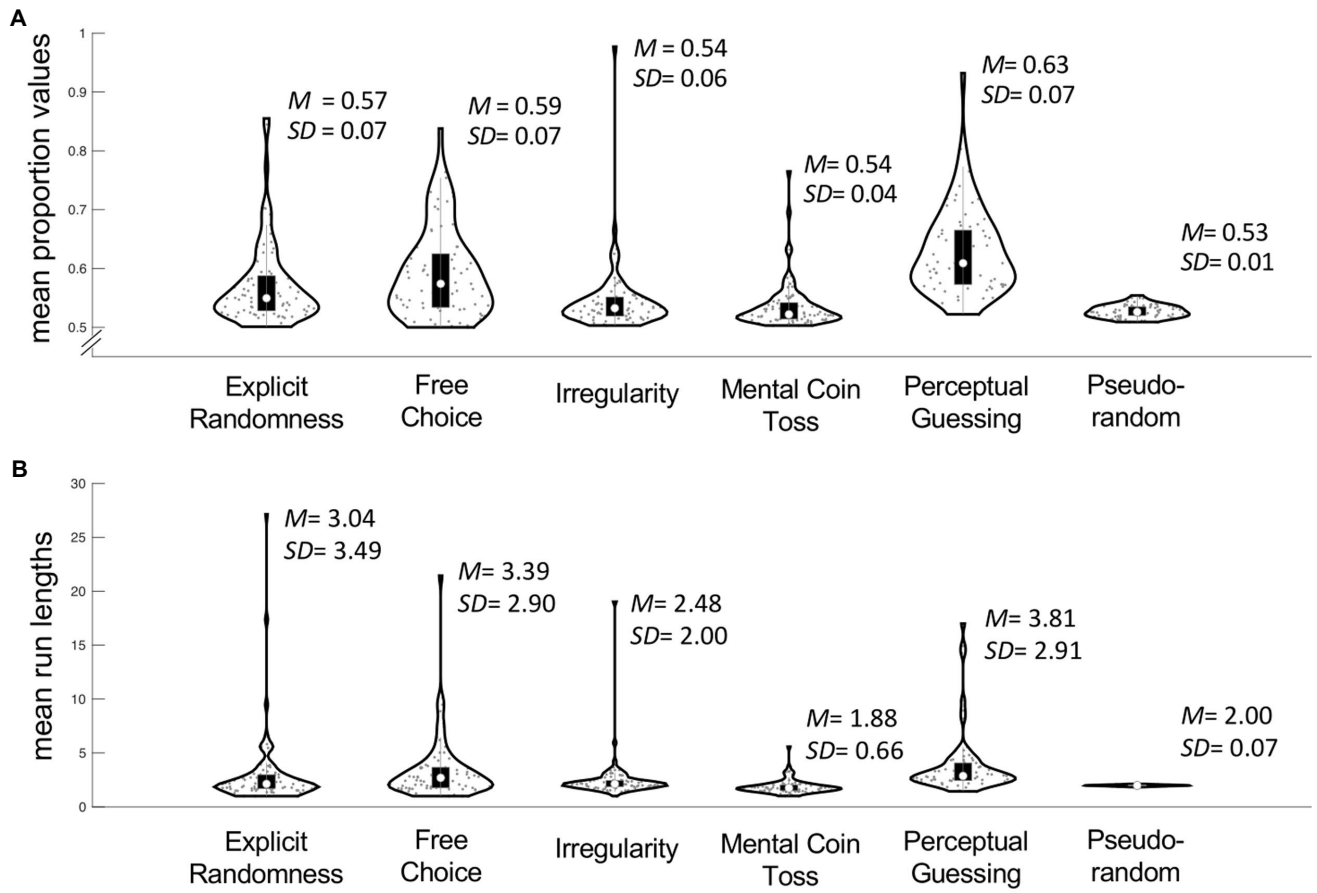
Overview of means of proportion values **(A)** and means of run lengths **(B)** averaged within subjects across five blocks and between subjects across five conditions. The outline of each violin plot shows the kernel density estimate, the black bars in the center indicate the interquartile range with the white dot representing the median.

In order to get a better understanding of the time series’ statistical properties we then calculated mean proportion values and run lengths for each participant (Figure 5). In terms of mean proportions, participants in *Irregularity* (IR; *M* = 0.54, *SD* = 0.06) and *Mental Coin Toss* (MC; *M* = 0.54, *SD* = 0.04) conditions had the most balanced sequences. These proportion values were closest to those of the pseudorandom sequences of 0.53 (*SD* = 0.01). With an average of 0.63 (*SD* = 0.07) the performance in *Perceptual Guessing* was the least balanced. The mean run length closest to those of the pseudorandom sequences (*M* = 2.00, *SD* = 0.07) was achieved in MC with a run length of 1.88 (*SD* = 0.66). Results in *Explicit Randomness* (ER), *Free Choice* (FC) and *Perceptual Guessing* (PG) show an average run length of around 3–4 (*M* = 3.04, *SD* = 3.49; *M* = 3.39, *SD* = 2. 90; *M* = 3.81, *SD* = 2.91 respectively).

### 7.3. Effect of instructions on entropy and optimal Markov order

Conditional entropy values and optimal Markov orders were averaged across the five blocks. The median and interquartile range of these averaged values are shown in Table 3. The multilevel model that was specified in the preregistration is reported in the Appendix, as it showed identical results to the following simpler Kruskal-Wallis test over the mean values. In an additional exploratory analysis, we extended our main preregistered analysis to include a set of pseudorandom sequences created by MATLAB’s pseudorandom generator as a sixth condition to illustrate the difference to the human generated sequences (see Appendix, Figure A3).

**Table 3:** Median (Med) and interquartile range (IQR) of conditional entropy values and optimal Markov orders per condition.

Condition	*n*	Conditional entropy Med IQR	Optimal Markov order Med IQR
Explicit Randomness (*ER*)	80	0.84 (0.25)	1.0 (0.80)
Free Choice (*FC*)	73	0.75 (0.34)	1.0 (0.80)
Irregularity (*IR*)	80	0.94 (0.10)	0.4 (0.80)
Mental Coin Toss (*MC*)	85	0.92 (0.11)	0.6 (0.80)
Perceptual Guessing (*PG*)	70	0.83 (0.15)	0.8 (0.60)

### 7.4. Conditional entropy

**Figure 6:**
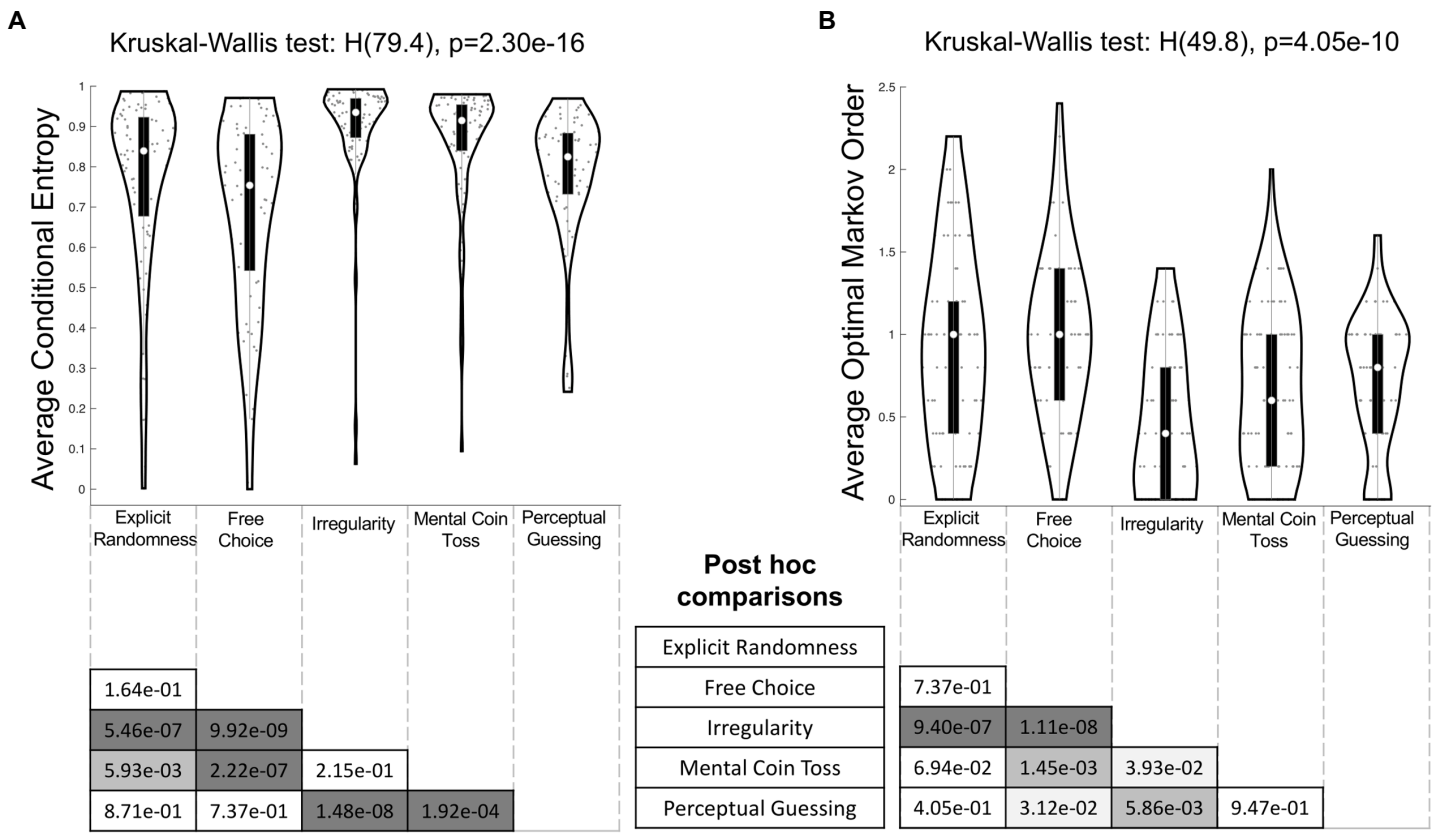
Tukey–Kramer adjusted *post hoc* comparisons of **(A)** average conditional entropy values and **(B)** optimal Markov orders between conditions. The outline of each violin plot shows the kernel density estimate, the black bars in the center indicate the interquartile range with the white dot representing the median. The *x*-axis labels of the violin plots serve as the columns for the half matrices in the bottom, so that each entry displays the *p* value of the comparison between the intersecting pair of row and column conditions. Color code of matrix entries: dark grey: *p* < 0.001, middle grey: *p* < 0.01, light grey: *p* < 0.05, white: not significant.

A one-way between-subjects Kruskal-Wallis test showed that conditional entropy values differed significantly between the conditions (H(79.4), *p* = 2.30e–16) with a large effect size $\eta^{2}$=0.20 95% CI [0.11, 0.26] (transformed to Cohen’s d = 0.99). Tukey–Kramer adjusted post-hoc tests yielded six significant pairwise comparisons (Figure 6A). Performance in both the *Irregularity* (*Med* = 0.94, *IQR* = 0.10) and *Mental Coin Toss* (*Med* = 0.92, *IQR* = 0.11) conditions differed significantly from the performance in the others conditions *Explicit Randomness* (*Med* = 0.84, *IQR* = 0.25, *p* = 5.46e-07 and *p* = 5.93e-03 respectively), *Free Choice* (*Med* = 0.75, *IQR* = 0.34 *p* = 9.92e-09 and *p* = 2.22e-07 respectively) and *Perceptual Guessing* (*Med* = 0.82, *IQR* = 0.15, *p* = 1.48e-08 and *p* = 1.92e-04 respectively).

### 7.5. Optimal Markov order

The results of a one-way Kruskal-Wallis test carried out on optimal Markov order shown in Figure 6B revealed a significant effect of condition (H(49.8), *p* = 4.05e–10) with an intermediate effect size $\eta^{2}$=0.12, 95% CI [0.04, 0.17] (transformed to Cohen’s d = 0.74). A post-hoc analysis with Tukey–Kramer adjustment showed that performance in *Irregularity* (*Med* = 0.4, *IQR* = 0.80) differed significantly from all other conditions. Additionally, *Free Choice* (*Med* = 1.0, *IQR* = 0.80) differed significantly from *Mental Coin Toss* (*Med* = 0.6, *IQR* = 0.80, *p* = 1.45e-03) and *Perceptual Guessing* (*Med* = 0.8, *IQR* = 0.60, *p* = 3.12e-02).

## 8. Discussion

The purpose of this study was to compare randomness in sequences across different instruction types. Our findings show that conditional entropy values in *Irregularity* (IR) and *Mental Coin Toss* (MC) conditions differed significantly from those in *Explicit Randomness* (ER), *Free Choice* (FC) and *Perceptual Guessing* (PG). In terms of optimal Markov order a similar finding for the IR condition was reported, as average Markov orders in this condition differed from all the rest. In general, we found that sequences were most random when people were either instructed to be “as irregular as possible” or to “mentally simulate a coin toss.” The least random performance for both outcome variables was found in the “free choice” (FC) sequences, lending further evidence to the existence of sequential dependencies in these types of tasks (see Allefeld et al., 2013; Lages et al., 2013 for a discussion). Explicitly instructing the participants to select randomly as well as performing perceptual guesses scored somewhere in the middle.

### 8.1. Irregularity and mental coin toss instructions produce the most random sequences

Asking to make irregular choices (IR) yielded the most random sequences. This contrasts with our initial prediction that these participants would show worse randomization results. We initially considered two possible mechanisms: First, we reasoned that during this task there was a higher working memory load, as the need to act irregularly would require actively monitoring and updating the choice history (which was not necessary in FC and PG tasks). A higher working memory load has been shown to affect randomization performance negatively (Naefgen and Janczyk, 2018). Second, according to ironic process theory the intention of controlling a mental state (e.g., suppressing a specific thought) is accompanied by error monitoring processes which undermine that very effort. This results in a higher likelihood of that thought to emerge, the opposite of what was intended (Wegner, 1994; Wang et al., 2020). Applying this line of reasoning to our experiment we predicted a worse randomization performance in the IR instructions, because they encouraged the suppression of patterns or regularities (as opposed to FC or PG instructions where suppression was not encouraged at all, see Table 1). Our findings do not support these predictions.

We propose the following possible explanations for the good randomization performance. First, the instructions could have been perceived as more intuitive and tied to known experiences. People have experience with behaving unpredictably, e.g., in competitive game settings (protean behavior). Second, this instruction is formulated in a way that implies a second agent (real or imaginary) who observes the sequence and tries to detect a pattern (see Table 1) which might facilitate randomness generation. Third, IR instruction might also simply be clearer and not evoke preconceived notions on the concept of randomness (Finke, 1984).

Similarly, simulating a coin toss in the head (MC) produced the second best randomization outcome. This is in agreement with the idea that focusing on the randomness of the process rather than the output should eliminate instructional biases (Brugger, 1997). It is possible that this instruction redirects the focus more to the process of tossing than to the resulting output, i.e., heads or tails. Further, according to a recent review by Schulze and Hertwig (2021), experiential engagement with a task versus a mere text-based description is an important predictor of statistical reasoning outcomes in infants and adults. The mental simulation of a coin toss is arguably a more experiential activity than for instance simply trying to be random. Consequently, imagining a toss would potentially evoke more visual and motor cortex activation. Another possible explanation that could be at play in the MC condition is the imagination effect. This phenomenon was first reported as an improvement in learning outcomes when students were encouraged to imagine a procedure to solve a problem as opposed to simply understanding and remembering it (Cooper et al., 2001). Hence, the mere act of imagination might have been a determining factor for the improved randomization performance in our experiment.

### 8.2. No statistical difference between explicit randomness and free choice tasks

We did not find any evidence for differences between ER and FC tasks in terms of optimal Markov orders and conditional entropy. This is particularly noteworthy as many experiments in the volition and action selection literature use free/spontaneous choice paradigms with the aim to essentially elicit a random response (Elsner and Hommel, 2001; Frith, 2013; Naefgen and Janczyk, 2018). Interestingly, even without requiring balanced sequences or avoiding patterns (as is typically done in these types of tasks) the FC behavior is similar to the ER condition. From this perspective it seems reasonable to use this type of task in volition experiments.

At the same time the lack of a significant difference is striking when viewed from a mechanistic perspective. In Baddeley et al.’s (1998) model (and later Cooper (2016)) a response is generated by first choosing from a set of schemas (i.e., choices options) and then evaluated against the choice history stored in the working memory. If deemed “random” enough it is applied, otherwise it is discarded and the process starts over. Applying this line of reasoning to the tasks, we expected both ER and FC tasks to include some form of choice (or schema) selection. However, it remains unclear what shape an evaluation process would take in the FC condition because here the instructions did not require the evaluation of the choice in a sequential context (as opposed to the ER task).

Perhaps individuals in the FC condition used their own criteria to evaluate their responses which were not explicitly mentioned in the task instructions. For instance, some people might have autonomously decided to balance their sequences (while others decided to exclusively press one button). This could explain the large spread of randomization measures in the FC condition (highest SD of the conditional entropy distribution). Response values in ER were also highly spread out which is not surprising given that large interindividual differences in randomization biases (such as the recency bias) are a consistent finding in the RSG literature (Wagenaar, 1972; Budescu, 1987; Shteingart and Loewenstein, 2016).

Our findings showed that the randomization performance in the FC task was among the least random (see Lages et al. (2013) and Allefeld et al. (2013) for a related discussion). This implies that there exist better ways to elicit random responses in future studies such as appealing to irregularity of a sequence or encouraging participants to perform a mental coin toss.

### 8.3. Incidental randomness in the perceptual guessing task

Similar to the FC condition, participants’ behavior in the PG condition was less random than in the other conditions. However, it is still remarkable that this experimental manipulation actually worked at all: 70 out of 132 participants thought that there were detectable differences between the identical tonal values. Participants’ answers before and after the debriefing reveal that they were genuinely surprised and in disbelief about the fact that the coins were identical, e.g.: “This was good fun and I really thought the coins were different in tonal values. I was surprised to learn they were not!” or “I made myself believe that there were different tonal values.” If there was no externally discriminable information to base the choice on, it stands to reason that any perceptual difference originated from events internal to the participant.

A potential explanation of why people believed to see a difference in tonal value between the coin images is that perception is not an objective representation of the outside world but is modulated by priors, higher-order predictions causing illusory percepts (Jolij et al., 2011; Gold and Stocker, 2017). In the PG instructions we emphasized the difficulty of the task and stated that we know from previous studies that people are actually able to identify the darker coin and make the right decision even if they think they do not see anything. This created expectations which likely shaped bottom-up visual processing to the extent that participants believed there was an actual difference in tonal value. This modulation can happen rather early, as Vilidaite et al. (2019) showed in a recent study on contrast discrimination where participants were required to make perceptual decisions between identical stimuli. The authors found that signals in the early visual pathway, presumably neural noise, already less than 100 ms after stimulus onset, contained information that was predictive of the upcoming choice.

The general modulatory top-down effect on expectation on visual perception provides a reason for why the manipulation worked at all. However, it does not explain the specific visual content that participants believed to see. Put differently, what were the mechanisms that led to any one of the two choices, heads or tails?

A potential contributing factor might be an underlying choice history bias, a well-established phenomenon in the perceptual decision-making literature. Here consecutive perceptual judgments on sequentially uncorrelated stimuli bear a degree of sequential (or serial) dependency, i.e., any given choice is influenced by the choices that came before it (Fischer and Whitney, 2014). This dependency can manifest itself as an over-alternation or over-repetition of choices within a sequence (Urai et al., 2019). The autocorrelation present in natural scenes acts as another prior, resulting in sequentially dependent perceptual decisions (Bliss et al., 2017; Urai et al., 2019). Importantly, serial dependency is positively affected by stimulus similarity, so the harder the ability to discriminate the more likely the observer will rely on choice history as a guide (Cicchini et al., 2018; Gallagher and Benton, 2022). In our data the tendency to repeat the previous choice is represented as the average run length, which is the highest in the PG condition (*M* = 3.81, *SD* = 2.91; Figure 5).

However, it is important to recognize that despite the sequential dependencies, the overall performance in the PG condition was still quite random (conditional entropy *Med* = 0.83, *SD* = 0.15 and optimal Markov Order *Med* = 0.8, *SD* = 0.6). Moreover, performance in PG was statistically indistinguishable from ER in terms of conditional entropy and optimal Markov orders. Essentially, even in the absence of any explicit suggestion of acting randomly, we still see an intrinsic element of randomness in the PG sequences, which remains after accounting for sequential dependency effects in visual perception. There have been several papers discussing the source of variability which could be either peripheral or central (Shadlen et al., 1996; Urai and Donner, 2022).

Interestingly, PG and FC conditions differed significantly only in terms of optimal Markov order, but not conditional entropy. This finding is partly comparable to Bode et al.’s (2013) results, where subjects also performed both a free choice and perceptual guessing task. In this study the perceptual guessing task required the participants to indicate whether a target object was a chair or piano, while in reality the presented stimulus contained no information (visual noise). The authors report that both tasks share the same neural substrates in the medial posterior parietal cortex and suggest that both task types potentially share similar mechanisms to produce internal choices. However, the comparison to Bode et al.’s (2013) results is limited, because the conditions were (1) administered within-subject as opposed to our between-subject design and (2) presented in random order, so that they did not record an uninterrupted choice sequence in each condition as was the case in our study.

### 8.4. Temporal stability of generated sequences

Our alternative multilevel analysis (see Appendix) showed that accounting for a potential block effect did not make a large difference in terms of the final results. Even though the block effect was significant for both outcome variables, it was very small. Considering that one third of participants indicated that they changed their approach at least once during the experiment, it is noteworthy that this did not translate into a large block effect. As a consequence, the multilevel results reported in the Appendix were identical to those of the simpler analysis, reported in the main text. This might support the idea that sequence characteristics are potentially stable across time. People tended to make decisions in a similar fashion, irrespective of the breaks in-between. This temporal inter-block stability is good news for research on using human random sequences as individual fingerprints or biometric verification devices (Jokar and Mikaili, 2012).

### 8.5. Limitations

We would like to point out limitations of this study that could be addressed in future research. First, the measures that we used to assess the choice sequences cannot capture all possible deviations and dependencies that might exist. The RSG literature in the past decades has used many different measures. However, the trend goes to abandoning the use of many separate indices and finding ways to adopt all-encompassing, parsimonious tests that capture the same information. Examples are algorithmic complexity approaches (Gauvrit et al., 2014), recurrence quantification analysis (Oomens et al., 2015) or Markov chain modeling – as was done in our study. While this limitation is in principle impossible to overcome, there is still much room for improvement in terms of creating a standardized procedure of randomness assessment of behavioral data.

Second, our results are based on binary choices. As mentioned before, a major problem in the RSG literature is the heterogeneity in task characteristics, one of which being different choice set sizes. It is not unlikely that cognitive processing is affected by whether one has to choose randomly between a large set of 0–9 digits or a binary set of 0–1. However, we believe that opting for a binary choice set in our paradigm was a sensible choice since a larger choice set would introduce a higher load on working memory. After all, our objective was to keep the task as simple as possible to focus on the main differences between different task instructions. Moreover, a larger choice set would make the Markov process-based analysis require much more data. The problem of different set sizes can be dealt with in future iterations of our experimental setup.

Third, an alternative approach to our research question could have been to use a within-subject design as it would allow for a stronger control for individual characteristics, which, as our data show, are somewhat heterogeneous. However, considering that we had a large sample size, it is safe to say that these concerns are kept within limits.

Finally, although we have implemented multiple attention checks to filter out inattentive participants, one might argue that the lack of performance-based payment (i.e., payment contingent on randomization performance) reduced the participants’ motivation to diligently make 1,000 binary choices and follow a particular instruction. Consequently, participants might instead have engaged in satisficing practices such as simply pressing left and right arrow keys on time, i.e., employing just enough effort to get paid. Note that this criticism is not specific to online studies but extends to laboratory-based experiments as well. The most direct tool at our disposal to probe this question was to repeatedly ask participants how closely they followed the instructions in every block. As shown in the results, 96.9% reported following the instructions “very closely” to “closely.” Also, as we have described above, it seems that there was no considerable decline in performance throughout the blocks, which provides reassurance that motivation levels remained at least unchanged. Additionally, we read through the written answers on the open-ended questions about participants’ strategy and the final general feedback and comments textbox. The overall impression is that participants appeared to be curious and interested about the experiment, perhaps because the tasks were unusual compared to the other experiments on this platform. Another key point to consider is a recent study by Schild et al. (2021) who reported a negative relation between approval rate and participant dishonesty. The mean approval rate (Prolific score) in our study was 99.4 (*SD =* 1.5) which following Schild et al.’s (2021) results would suggest lower cheating or satisficing behavior.

At the same time, an incentivized experiment with a reward schedule would probably have a strong impact on randomization performance, as (Neuringer, 2002) showed in a wide range of experiments. However, this is a tricky setup given the elusive conceptualization of randomness – the conceptual difficulty is to decide on how to measure the desired randomization behavior unambiguously. An intriguing design for future experiments would be to simply inform the participant that their payment will depend on their performance, when in reality everyone would be paid the same in the end. Another motivation-increasing factor would be to introduce a game scenario with an actual or computer opponent, as has been done several times (Lee and Seo, 2011; Wong et al., 2021).

### 8.6. Outlook

In this study we have shown that randomness in generating sequences differs based on how it is instructed. The next crucial step is to understand why these differences exist. We have described potential reasons for why MC and IR were so successful, so further research is required to disentangle these different explanations. By understanding the necessary components which determine the good performance we can devise new and improved ways of eliciting random decision-making. At the same time, it would allow for deeper insights of the cognitive processes involved in the production of randomness in general.

Furthermore, an interesting question for future research is whether the randomness is dependent on whether participants think about their decisions in either a motor (or input) based framework or a sensory (or output) based framework (Janczyk et al., 2020).

In terms of Marr’s three level framework (Marr, 1982) a deep dive in the me implementational level could provide interesting insights on how the different randomization tasks are implemented on a physical level. Contemporary research on the neural bases of randomization is surprisingly limited and could be a valuable area to explore given the newly available analysis tools such as multivariate decoding techniques of neuroimaging data. For example, investigating brain activity by examining the role of the dorsolateral prefrontal cortex (DLPFC) in the context of the network modulation model (Jahanshahi et al., 2000). In this model the left DLPFC suppresses the spread of activation in in the number associative network in the superior temporal cortex (STC) to inhibit habitual counting.

Another emerging field that has not yet been brought in explicit connection with RSG in particular is the area of neural variability research. Of special interest would be to look into its role in terms of inter-individual trait as well as intra-individual state differences in an RSG task (Waschke et al., 2021). Not only did our data show that participants perform a randomization task differentially depending on instruction, it also revealed a high amount of individual variation. The key questions are whether and how individuals can up-and downregulate behavioral variability and if they potentially leverage neural variability signals. Waschke et al. (2021) call this modulatory ability “meta-variability,” which is a similar idea found in the behavioral literature introduced by (Neuringer, 2002) as described in the introduction. The author stipulates an organism’s overarching capacity to impose different levels of variability based on different environmental demands. Whether this calibration of variability is subject to conscious control is another important question to investigate in future studies as it might be useful for improving cognitive control and regulation in clinical populations characterized by variability deficiencies.

## 9. Conclusion

Our study demonstrates that the instructions in random sequence generation tasks influence the randomness of performance. The most random sequences were created in the tasks where participants were instructed to choose irregularly and where they had to mentally simulate a coin toss. This suggests that special care should be given to the exact task instructions and that this latent effect might have contributed to the heterogeneity in the literature.

## Data availability statement

The datasets presented in this study can be found in online repositories. The names of the repository/repositories and accession number (s) can be found at: https://osf.io/z8rjx/files.

## Ethics statement

The studies involving human participants were reviewed and approved by Ethikkommission - Institut für Psychologie, Humboldt-Universität zu Berlin. The patients/participants provided their written informed consent to participate in this study.

## Author contributions

MG, CB, and J-DH contributed to the conception and design of the study. MG created and executed the study, conducted the analyses and drafted the manuscript. CA contributed by providing insights and guidance on how to measure randomness and conduct the behavioral analyses. He also wrote section 6.4.3 on the randomness measures. All authors contributed to the article and approved the submitted version.

## Funding

This work was funded by the Excellence Initiative of the German Federal Ministry of Education and Research (Excellence Cluster science of Intelligence) and the Max Planck School of Cognition.

## Conflict of interest

The authors declare that the research was conducted in the absence of any commercial or financial relationships that could be construed as a potential conflict of interest.

## Publisher’s note

All claims expressed in this article are solely those of the authors and do not necessarily represent those of their affiliated organizations, or those of the publisher, the editors and the reviewers. Any product that may be evaluated in this article, or claim that may be made by its manufacturer, is not guaranteed or endorsed by the publisher.

## Footnotes

^1^ www.prolific.co
