# Supplementary material for: Instruction effects on randomness in sequence generation
Source: Front Psychol. 2023 Mar 22;14:1113654. doi: 10.3389/fpsyg.2023.1113654 (PMC10075230; doi:10.3389/fpsyg.2023.1113654)
Supplement: Supplementary file 1 [file Data_Sheet_1.pdf]

1 Appendix

1.1 Assessment of block effects with mixed-effects model

Since we suspected that there might be performance differences that would occur in the course of the five blocks, we analyzed the data by a multilevel approach. As shown in Figure A1 (bottom) the mixed-effects model in the first level was chosen to eliminate potential block effects.

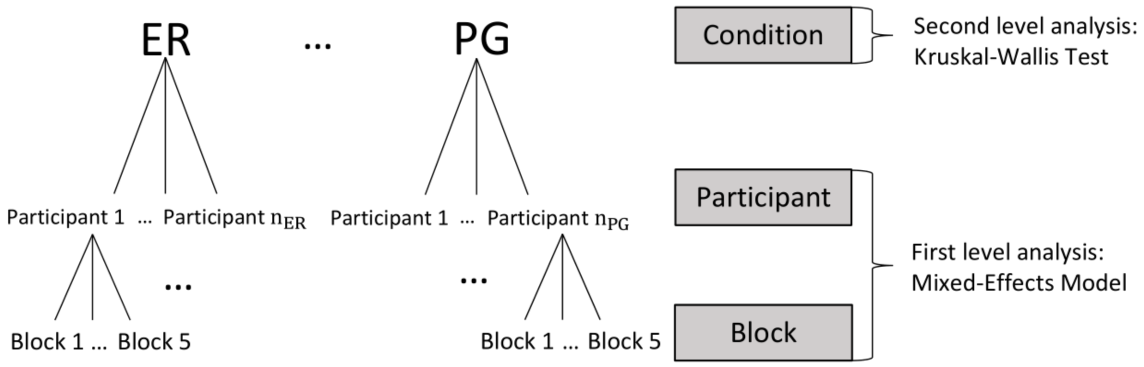

Figure A1. Diagram of nested data structure.

In the linear mixed-effects model each outcome variable was explained by block number (centered, i.e., -2 to 2) as fixed and random effect plus random intercept.

1. $\text{Conditional Entropy}_{ij} = \beta_0 + \beta_1 \text{centeredBlock}_{ij} + b_{0i} + b_{1i}\text{centeredBlock}_{ij} + e_{ij}$

2. $\text{Optimal Markov Order}_{ij} = \beta_0 + \beta_1 \text{centeredBlock}_{ij} + b_{0i} + b_{1i}\text{centeredBlock}_{ij} + e_{ij}$,

where i = subject (1…388) and j = Block (1…5). The parameters were estimated with the Maximum Likelihood estimation method. The covariance matrix was fully parameterized. Table A1 shows the results of this mixed-effects model. The fixed effect of *Block* is significant (p<0.001), but surprisingly very small for both conditional entropy (-0.01) and optimal Markov order (0.04). The standard deviation of the individual slopes (0.028 and 0.092) is also rather small which means that the block effect affected everybody in more or less similar ways.

Table A1. Linear mixed-effects model output

Dependent Variable	Conditional Entropy				Optimal Markov Order			
Fixed Effects								
	Estimate	SE	t	p	Estimate	SE	t	p
Intercept	0.799	0.010	76.613	0	0.746	0.026	28.310	7.479e-148
Block (centered)	-0.012	0.002	-5.894	4.432e-09	0.038	0.009	4.080	4.689e-05
Random Effects								
Groups: Subject	SD				SD			
Intercept	0.201				0.467			
Block (centered)	0.028				0.092			
Residual	0.092				0.508			
Model statistics								
	AIC	BIC	Log-likelihood	Deviance	AIC	BIC	Log-likelihood	Deviance
	-2244.7	-2211.3	1128.3	-2256.7	3644.4	3677.8	-1816.2	3632.4
	n observations= 1940,1 $R^2 = 0.867$, $R_a^2 = 0.867$				*n observations*= 1940, $R^2 = 0.474$, $R_a^2 = 0.473$			

Variability in random intercepts was quite large (SD=0.201 and 0.467) which indicates that performance in an "average" block (here where *centeredBlock* = 0, or equivalently *Block* = 3) differed a lot between individuals. To further quantify this, we calculated the intraclass correlation (ICC) values (between-subject variability/total variability). For the conditional entropy and optimal Markov order analyses ICC values were 0.83 and 0.48 respectively for the specified model, and 0.79 and 0.43 for the null model. This is not surprising as our model

has not taken into account that the participants were in different instruction groups, which is done in the next analysis (Fig A1 top). The relatively small difference between the ICCs in the specified vs. null model further indicates that the impact of introducing *Block* to the model was small. However, as this analysis was laid out in the preregistration, we still report the results here.

From this mixed-effects model we extracted the subject-wise random intercepts which then served as the dependent variable on the second level analysis. Table A2 shows the mean and spread of the intercepts for both outcome variables.

Table A2. Statistical characteristics of random intercepts

Condition	*n*	*Conditional Entropy* *M SD*	*Optimal Markov Order* *M SD*
Explicit Randomness (*ER)*	80	0.77 (0.22)	0.88 (0.47)
Free Choice *(FC)*	73	0.69 (0.23)	0.95 (0.41)
Irregularity *(IR)*	80	0.89 (0.14)	0.51 (0.36)
Mental Coin Toss *(MC)*	85	0.86 (0.15)	0.68 (0.38)
Perceptual Guessing *(PG)*	70	0.76 (0.17)	0.73 (0.33)

Here a one-way Kruskal-Wallis test was used to identify the differences in intercepts between the five instruction conditions (second level analysis Figure A1 top). This was done separately for optimal Markov order and conditional entropy values. Fig. A2 shows the distribution and Tukey-Kramer adjusted post hoc comparisons of the random intercepts that were extracted from regressions in the first level. The results are near identical to the main analysis which was based on the raw values of the outcome variables: The random intercepts based on conditional entropy values differed significantly between the conditions (H(78.96), p = 2.90e-16) with a large effect size η^2=0.20 95% CI [0.11, 0.27] (transformed to Cohen's d=0.99). Similarly, the Kruskal-Wallis test on random intercepts based on optimal Markov order showed a significant difference (H(50.75), p = 2.52e-10), with an intermediate effect size η^2=0.12 95% CI [0.04, 0.18] (transformed to Cohen's d=0.744). The Tukey-Kramer adjusted post hoc comparisons show the same pairwise differences as the main analysis with the same significance levels.

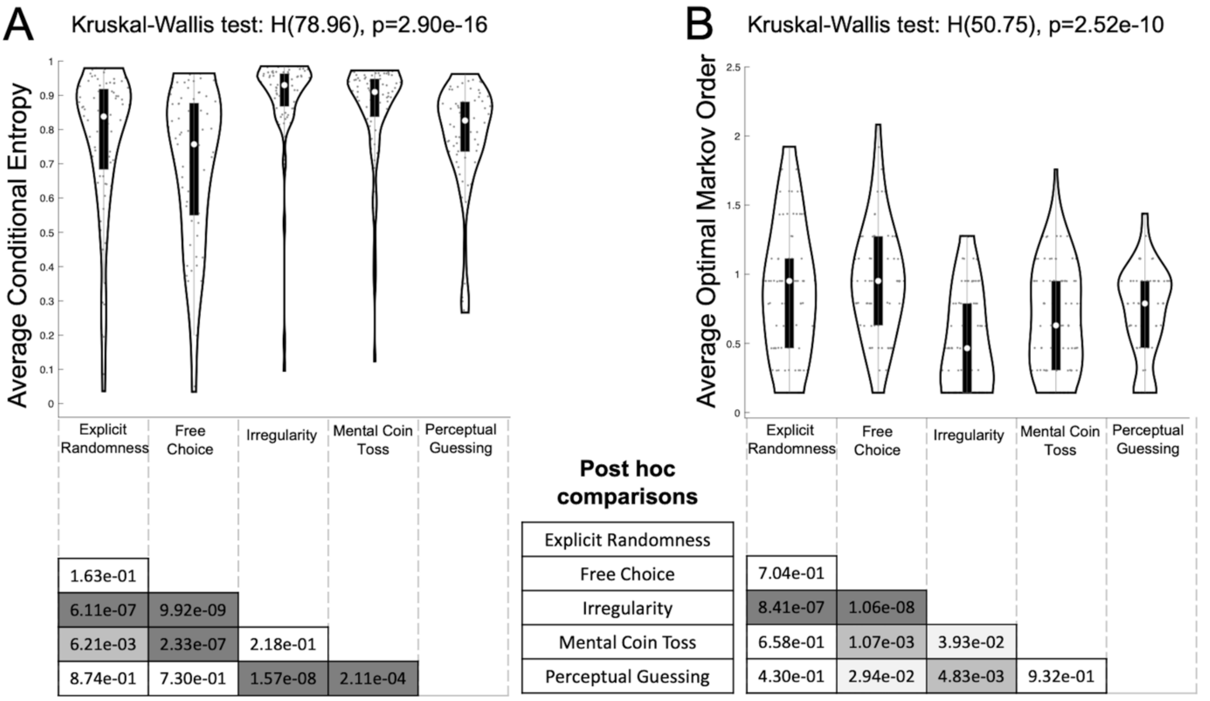

Figure A2. Tukey-Kramer adjusted post hoc comparisons of random intercepts from first level regressions based on (A) conditional entropy values and (B) optimal Markov orders between conditions. The x-axis labels of the violin plots serve as the columns for the half matrices in the bottom, so that each entry displays the p value of the comparison between the intersecting pair of row and column conditions. Color code of matrix entries: dark grey: $p < 0.001$, middle grey: $p < 0.01$, light grey: $p < 0.05$, white: not significant.

1.2 Extended analysis with simulated pseudorandom sequences

In an additional analysis we expanded the Kruskal-Wallis test and pairwise comparisons by including a set of 78 pseudorandom sequences of length 1000 using Matlab's "Mersenne twister" algorithm as a sixth condition to demonstrate the difference between human and computer generated sequences.

The Kruskal-Wallis test was significant for both randomness measures, (H(237.8), p=2.30e-49, eta^2=0.51 95% CI [0.44, 0.57]) for conditional entropy and (H(189.9), p=4.07e-39, eta^2=0.40, 95% CI [0.33,0.46]) for Markov order. Since we have added a sixth condition, the number of comparisons increased from 10 to 15 as compared to the main analysis (Figure A3) which made the p-values more conservative. As a result, the surviving Tukey-Kramer adjusted pairwise comparisons were the following:

In terms of conditional entropy, Irregularity (Med = 0.94, IQR = 0.10) differed significantly from Explicit Randomness (Med = 0.84, IQR = 0.25, p= 2.35e-05), Free Choice (Med = 0.75, IQR = 0.34, p=2.12e-08) and Perceptual Guessing (Med = 0.82, IQR = 0.15, p=6.21e-07). Mental Coin Toss (Med = 0.92, IQR = 0.11) differed significantly from Perceptual Guessing (p=0.005) and Free Choice (p=4.37e-07). Additionally, all conditions differed significantly from the pseudorandomly generated sequence (Med = 0.99, IQR=0.001) with $p < 0.001$.

In terms of Markov order, Irregularity (Med = 0.4, IQR = 0.80) differed significantly from Explicit Randomness (Med=1, IQR=0.80, p=7.87e-06), Free Choice (Med=1, IQR=0.80, p=5.14e-08) and Perceptual Guessing (Med=0.8, IQR=0.60, p= 0.015). Also, Free Choice (Med = 1.0, IQR = 0.80) differed significantly from Mental Coin Toss (Med = 0.6, IQR = 0.80, p = 0.010). The pseudorandom sequence (Med = 0, IQR=0) differed significantly from all other conditions at p < 0.001.

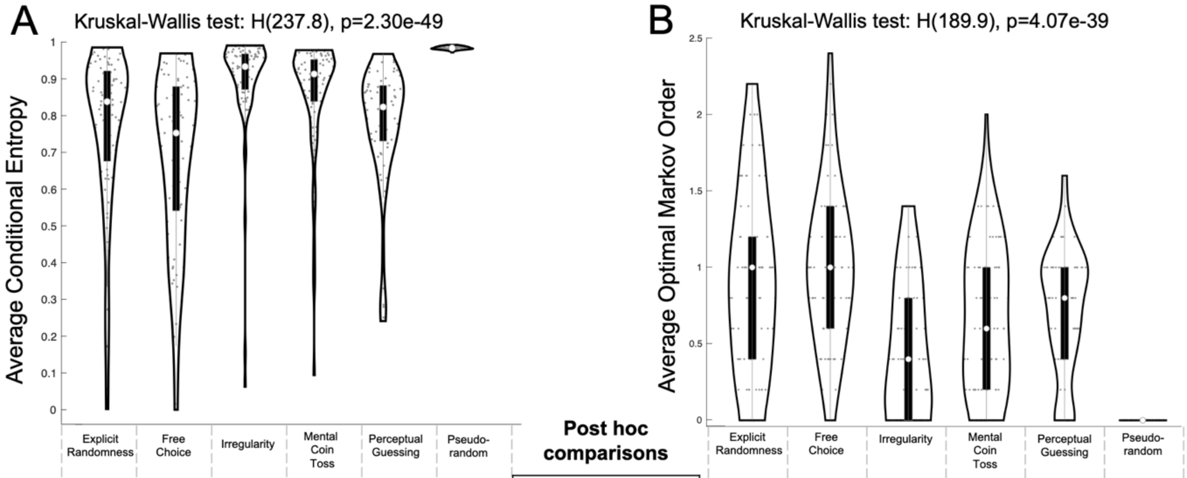

A

Post hoc comparisons	Explicit Randomness	Free Choice	Irregularity	Mental Coin Toss	Perceptual Guessing
Free Choice	0.392				
Irregularity	2.35e-05	2.12e-08			
Mental Coin Toss	0.060	4.37e-05	0.300		
Perceptual Guessing	0.958	0.911	6.21e-07	0.005	
Pseudorandom	2.07e-08	2.07e-08	2.10e-08	2.07e-08	2.07e-08

B

Post hoc comparisons	Explicit Randomness	Free Choice	Irregularity	Mental Coin Toss	Perceptual Guessing
Free Choice	0.885				
Irregularity	7.87e-06	5.14e-08			
Mental Coin Toss	0.189	0.01	0.061		
Perceptual Guessing	0.591	0.09	0.015	0.991	
Pseudorandom	2.07e-08	2.07e-08	3.76e-08	2.07e-08	2.07e-08

Figure A3. Tukey-Kramer adjusted post hoc comparisons of (A) average conditional entropy values and (B) optimal Markov orders between conditions. The outline of each violin plot shows the kernel density estimate, the black bars in the center indicate the interquartile range with the white dot representing the median. The x-axis labels of the violin plots serve as the columns for the half matrices in the bottom, so that each entry displays the p value of the comparison between the intersecting pair of row and column conditions. Color code of matrix entries: dark grey: p < 0.001, middle grey: p < 0.01, light grey: p < 0.05, white: not significant.
